# Bio-inspired Machine Learning for Distributed Confidential Multi-Portfolio Selection Problem

**DOI:** 10.3390/biomimetics7030124

**Published:** 2022-08-29

**Authors:** Ameer Tamoor Khan, Xinwei Cao, Bolin Liao, Adam Francis

**Affiliations:** 1 Department of Computing, The Hong Kong Polytechnic University, Hong Kong 999077, China; 2 School of Business, Jiangnan University, Wuxi 213031, China; 3 College of Computer Science and Engineering, Jishou University, Jishou 409811, China; 4 Faculty of Science and Engineering, Swansea University, Swansea SA1 8EN, UK

**Keywords:** multi-portfolio, optimization, swarm algorithm, beetle antennae search, stochastic algorithm, distributed beetle antennae search, investment, stocks

## Abstract

The recently emerging multi-portfolio selection problem lacks a proper framework to ensure that client privacy and database secrecy remain intact. Since privacy is of major concern these days, in this paper, we propose a variant of Beetle Antennae Search (BAS) known as Distributed Beetle Antennae Search (DBAS) to optimize multi-portfolio selection problems without violating the privacy of individual portfolios. DBAS is a swarm-based optimization algorithm that solely shares the gradients of portfolios among the swarm without sharing private data or portfolio stock information. DBAS is a hybrid framework, and it inherits the swarm-like nature of the Particle Swarm Optimization (PSO) algorithm with the BAS updating criteria. It ensures a robust and fast optimization of the multi-portfolio selection problem whilst keeping the privacy and secrecy of each portfolio intact. Since multi-portfolio selection problems are a recent direction for the field, no work has been done concerning the privacy of the database nor the privacy of stock information of individual portfolios. To test the robustness of DBAS, simulations were conducted consisting of *four* categories of multi-portfolio problems, where in each category, *three* portfolios were selected. To achieve this, 200 days worth of real-world stock data were utilized from 25 NASDAQ stock companies. The simulation results prove that DBAS not only ensures portfolio privacy but is also efficient and robust in selecting optimal portfolios.

## 1. Introduction

Portfolio optimization is a hot research topic in academia, since it enables investors to make an optimal decision between profit and risk. This refers to the method of making the best investment in numerous stocks [1]. Apart from this, there are several other real-world constraints that researchers have taken care of over time, for instance, cardinality constraint, tax-aware constraint, lower and upper bounds, multiple portfolios, round-lot constraint, stock size, computational and time complexities.

There are several state-of-the-art algorithms that have been proposed over time to tackle these constraints. For instance, an unconstrained portfolio optimization problem can be easily solved using linear or quadratic programming, but constraints make it a complex optimization problem to solve. Ref. [2] showed that the simple addition of a cardinality constraint makes it an NP-complete problem. Therefore, there is no straightforward algorithm to compute the exact optimality of the problem. Techniques that use machine learning algorithms, heuristic algorithms, and black-box optimization are good alternatives to solve such highly complex, computationally and time-consuming problems. Ref. [3] proposed using multiple machine learning modules, i.e., coordinate descent, the proximal gradient, Dykstra, and alternating direction of multipliers. Their algorithm also accounts for several constrained portfolios, i.e., equal risk contribution, diversified, and risk budgeting portfolios. Likewise, Ref. [4] combined several portfolio models, i.e., equal-weighted modeling (EQ), mean-variance model, and Monte Carlo simulation modeling. Furthermore, to improve the portfolio problem based on its time-series nature, they applied a long short-term memory (LSTM) model. However, the major drawback of these methods is the training of hundreds of hyper-parameters, which makes them computationally expensive and slow to compute.

**Figure 1:**
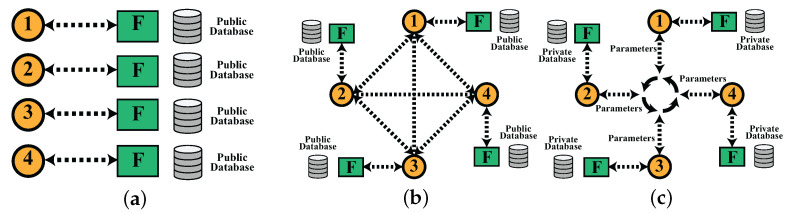
(**a**) shows the local learning where each BAS algorithm is assigned to one portfolio, and the objective is to optimize the respective portfolio. (**b**) shows the swarm architecture, where the swarm has access to a public database, where particles coordinate to obtain an optimal solution without considering the privacy of the database. (**c**) shows the distributed architecture, where particles only share the parameters, i.e., gradients, instead of the private information of the portfolios, i.e., private database, stock information, and client information.

In this paper, we are focused on the multi-portfolio selection problem while considering the investors’ privacy. Since the multi-portfolio problem has only recently gained attention from academia and financial practitioners, no work has been conducted regarding the confidentiality and the secrecy of private databases and individual portfolios. The multi-portfolio selection problem enables the optimization of tens of hundreds of portfolios simultaneously, making it computationally economical and time efficient. There are several proposed methods for multi-portfolio optimization in the literature [5,6,7,8,9,10,11,12,13,14,15,16,17]. The authors of [18,19] were the pioneers of the multi-portfolio optimization domain. The objective was to maximize social welfare, which was the sum of the utility of the individual accounts. For instance, ref. [20] proposed a hybrid model that includes a heuristic and combinatorial framework and solved the multi-portfolio problem under a risk–budget constraint. Ref. [21] proposed a Cournot–Nash equilibrium framework to solve the multi-portfolio problem. The major drawback being that each portfolio was treated individually, assuming that the others gave the best response. Ref. [22] overcame this issue by proposing a joint optimization problem, where the model was able to optimize the portfolio as well as cost splitting among the portfolios. Ref. [23] employed an information pooling game mechanism for the multi-portfolio optimization. Ref. [24] added a risk measurement constraint along with the selection of multi-portfolios. Ref. [25] proposed fairness-aware multi-participant satisfying (FMS) criterion to model a target-oriented strategy which optimized client portfolios by maximizing the returns. The major drawback of these techniques is the lack of privacy for the investors. They also solve the multi-portfolio problem, collectively making asset data exposed, meaning data are not secure or confidential. In order to provide privacy to each investor, i.e., portfolios regarding their investment and portfolio selection, it is necessary to design a distributed framework that optimizes each portfolio locally and optimizes all portfolios collectively. The three types of optimizing models are shown in Figure 1. It shows that in local learning, all portfolios are optimized individually, which is computationally and time-wise inefficient. Likewise, typical swarm learning models do not account for the confidentiality of the data and portfolio, and the particles share critical information about the portfolios to reach the optimal solution. However, in the distributed system, each particle deals with a single portfolio locally, and the particles share the objective function value, i.e., gradients alone to each other. Thus, they efficiently optimize the multi-portfolio selection problem without violating privacy.

In this paper, we propose a swarm variant of a known meta-heuristic algorithm known as Beetle Antennae Search (BAS). It mimics the food-collecting nature of the beetle in order to search for the optimal solution to a problem. BAS is a single particle searching algorithm, where the particle optimizes an objective function by searching the search space iteratively. The utility of BAS has expanded to several real-world problems [26,27,28,29,30,31,32,33,34,35,36,37,38,39,40,41,42,43,44,45,46,47,48,49], including the portfolio optimization. Ref. [50] employed BAS for the selection of the optimal portfolio under a non-convex cardinality constraint. Ref. [51] proposed a QBAS (Quantum Beetle Antennae Search) variant of BAS and solved the portfolio selection problem under cardinality constraint. Ref. [52] solved the time-varying portfolio selection problem under the transaction cost. Ref. [53] used the mean-variance model of portfolio optimization under two constraints: cardinality and transaction cost constraints. Ref. [54] proposed a hybrid framework of BAS-PSO and used it for portfolio optimization. From these research works, we can see the utility of BAS in portfolio optimization under real-world constraints. However, BAS has never been employed for the multi-portfolio optimization problem because of computational limitations. As mentioned earlier, BAS is a single particle searching algorithm, meaning it would be computationally challenging for BAS to optimize even a single portfolio with over 100 stock companies. Therefore, the efficiency of BAS will drop further if applied to the multi-portfolio selection problem.

We have proposed a swarm or distributed variant of BAS known as DBAS (Distributed Beetle Antennae Search). It is a hybrid variant with the swarm-like nature of Particle Swarm Optimization (PSO) and the BAS updating criteria. Each particle in DBAS will optimize a single portfolio, and collectively, the swarm will optimize all the portfolios without violating portfolio privacy. The DBAS will optimize the multi-portfolio in two stages by optimizing each portfolio locally and then optimizing all the portfolios globally without sharing any private data among portfolios. To the best of our knowledge, no researcher has considered the privacy issue while solving the multi-portfolio problem, since it is a newly emerging portfolio selection challenge. Through our proposed framework, we will optimize the multi-portfolio problem efficiently with low computation and time cost whilst ensuring the privacy of clients and their portfolios.

The rest of the paper is structured as follows: In Section 2, we will formulate the portfolio optimization problem. In Section 3, we will elaborate on the nature of BAS and will drive the DBAS variant. In Section 4, we will discuss the simulation results on a multi-portfolio selection problem with real-world stock data. In Section 5, we will conclude the paper with final remarks.

## 2. Problem Formulation

In this section, we will discuss the building blocks of the portfolio optimization problem. Later, we will elaborate on different portfolio models and select one for the DBAS algorithm. All the hyper-parameters are mentioned in Table 1.

### 2.1. Building Blocks of Portfolio Selection

Here, we will discuss the building blocks of portfolio selection, i.e., expected return (profit), risk, total investment, total transaction cost, and cardinality constraint.

#### 2.1.1. Expected Return

The primary factor in portfolio optimization is the maximization of expected return: in other words, the profit that investors are expecting from their investment in stocks. Let us say there are *K* total stocks and the normalized investment in all *K* stocks is given as $E=\left[E_{1},E_{2},E_{3},\cdots ,E_{i},\cdots E_{K}\right]\in R^{K},$ where *i* is the *i*-th stock and $E_{i}$ is the the normalized investment in the *i*-th stock. Now, let us say that the mean return of *K* stocks based on their past data is given as, $\epsilon ={\left[\epsilon_{1},\epsilon_{2},\epsilon_{3},\cdots ,\epsilon_{i},\cdots ,\epsilon_{K}\right]}^{T}\in R^{K}.$ The objective is to find the optimal E such that it maximizes the expected return, which is given as

(1) $$\begin{array}{c} \max_{E}\xi \left(E\right)=E\epsilon^{T}. \end{array}$$

To further consolidate (1) and to ensure that the expected return is above a certain threshold k, the modification to (1) is given as,

(2) $$\begin{array}{c} \max_{E}\xi \left(E\right)=E\epsilon^{T}-k\geq 0. \end{array}$$

here, *k* is a scalar quantity and $E\epsilon^{T}-k=0$ means the break-even point (no profit-loss).

#### 2.1.2. Risk

The maximization of profit (expected return) does not avoid the risk factor. The second objective is to minimize the risk. The risk minimization is given as,

(3) $$\begin{array}{c} \min_{E}\upsilon \left(E\right)={\mathrm{EQE}}^{T}, \end{array}$$

where Q is a covariance matrix, and it is given as,

(4) $$\begin{array}{c} Q=\left[\begin{array}{ccccc} Q_{11} & Q_{12} & Q_{13} & \cdots & Q_{1j}\\ Q_{21} & Q_{22} & Q_{23} & \cdots & Q_{2j}\\ Q_{31} & Q_{32} & Q_{33} & \cdots & Q_{3j}\\ \vdots & \vdots & \vdots & \vdots \cdots & \vdots \\ Q_{i1} & Q_{i2} & Q_{i3} & \cdots & Q_{ij} \end{array}\right], \end{array}$$

where $Q_{ij}$ is the variance of the *i*-th stock with *j*-th stock in the portfolio.

#### 2.1.3. Total Investment

Now, we will discuss one of the primary constraints: total investment. It ensures the allocation of the total asset in the stocks, and it is given as,

(5) $$\begin{array}{c} 1E^{T}=1, \end{array}$$

where 1 is a unit vector. As mentioned above, $E^{T}$ is a normalized investment, so the sum of the amount invested in the portfolio should be 1 as shown in (5). To ensure this, we need an additional constraint which is given as,

(6) $$\begin{array}{c} 0\leq E\leq 1. \end{array}$$

It will ensure that E remains positive and within [0,1].

#### 2.1.4. Total Transaction Cost

The total transaction cost is another essential constraint in portfolio selection. The transaction cost is positive if the investor buys stock and is negative if the investor sells stock. Let us say that Φ(E) represents the transaction cost of the portfolio; it is given as,

(7) $$\begin{array}{c} \Phi \left(E\right)=[\Phi_{1}\left(E_{1}\right),\Phi_{2}\left(E_{2}\right),\Phi_{3}\left(E_{3}\right),\cdots ,\Phi_{i}\left(E_{i}\right)], \end{array}$$

where i∈{1,2,3,⋯,K} and $\Phi_{i}\left(E_{i}\right)$ represent the transaction cost of the *i*-th stock. The total transaction cost refers to the sum of all the individual stocks, which is given as

(8) $$\begin{array}{c} \Phi_{t}\left(E\right)=\sum_{i=1}^{K}\Phi_{i}\left(E_{i}\right). \end{array}$$

If $\Phi_{t}\left(E\right)>0,$ it means more stock is bought than sold. Likewise, if $\Phi_{t}\left(E\right)<0,$ it means that more stock is sold than bought. Generally, the transaction cost includes some additional charges, which we can formulate using a linear transaction model [55], which includes a linear function $\alpha =[\alpha_{1},\alpha_{2},\alpha_{3},\cdots ,\alpha_{i}],$ where i∈{1,2,3,⋯,K}. The transaction cost of the *i*-th stock in the portfolio is given as,

(9) $$\begin{array}{c} \Phi_{i}\left(E_{i}\right)=\alpha_{i}E_{i}. \end{array}$$

We can rewrite (7) using (9) as,

(10) $$\begin{array}{c} \Phi \left(E\right)=\alpha E^{T}. \end{array}$$

We can combine (5) and (10) into a single constraint, which is given as,

(11) $$\begin{array}{c} \chi \left(E\right)=\left(1+\alpha \right)E^{T}=1. \end{array}$$

#### 2.1.5. Cardinality Constraint

Another real-world constraint in portfolio selection is known as the cardinality constraint. So far, we have assumed that investment is made in all of the *K* stocks. However, in a real case scenario, it is observed that the investor may want to exclude some stocks n(<K) from the final portfolio selection, which is known as a cardinality constraint. We can make such a decision by introducing a binary decision vector, i.e., $b=[b_{1},b_{2},b_{3},\cdots ,b_{i},\cdots ,b_{n}],$ where $b_{i}\in \left\{0,1\right\}.$ According to the cardinality constraint,

(12) $$\begin{array}{c} \sum_{i=1}^{K}b_{i}=n. \end{array}$$

We also need to reformulate (6) according to (12), which is given as,

(13) $$\begin{array}{c} 0\leq E\leq b. \end{array}$$

### 2.2. Portfolio Selection Models

Here, we will discuss the three known models for portfolio selection: mean variance model, efficient frontier model, and Sharpe ratio model.

#### 2.2.1. Mean-Variance Model

The primary objective of the mean variance model is to minimize the risk involved in portfolio selection, and it treats the expected return (profit) as a constraint. We can formulate the mean variance model using (2), (3), and (11)–(13), which is given as,

$$\begin{array}{cc} \mathrm{Minimize}:\; & \end{array}$$

(14) $$\begin{array}{cc} \; & \min_{E}\upsilon \left(E\right)\\ \mathrm{Subject}\;\mathrm{to}:\; & \end{array}$$

(15) $$\begin{array}{cc} \; & \xi \left(E\right)=E\epsilon^{T}-k\geq 0 \end{array}$$

(16) $$\begin{array}{cc} \; & \chi \left(E\right)=\left(1+\alpha \right)E^{T}=1 \end{array}$$

(17) $$\begin{array}{cc} \; & \sum_{i=1}^{K}b_{i}=n \end{array}$$

(18) $$\begin{array}{cc} \; & 0\leq E\leq b. \end{array}$$

The mean variance model is not very flexible, since it restrains the growth in profits. To overcome it, we can make use of an efficient frontier model.

#### 2.2.2. Efficient Frontier Model

In this model, we include expected return (profit) in the objective function. We can formulate the frontier model using (2), (3), and (11)–(13), which is given as,

$$\begin{array}{cc} \mathrm{Minimize}:\; & \end{array}$$

(19) $$\begin{array}{cc} \; & \min_{E}\lambda \upsilon \left(E\right)-\left(1-\lambda \right)\xi \left(E\right)\\ \mathrm{Subject}\;\mathrm{to}:\; & \end{array}$$

(20) $$\begin{array}{cc} \; & \chi \left(E\right)=\left(1+\alpha \right)E^{T}=1 \end{array}$$

(21) $$\begin{array}{cc} \; & \sum_{i=1}^{K}b_{i}=n \end{array}$$

(22) $$\begin{array}{cc} \; & 0\leq E\leq b. \end{array}$$

here, λ∈[0,1]. If λ=0, then the model will maximize the expected-return (profit). However, if λ=1, then the model will minimize the risk. The value of λ between [0,1] will be a trade-off between profit and the risk. The only drawback of the model is that it involves the use of a hyper-parameter λ. To avoid this, we will look into a Sharpe model, which we will optimize using DBAS.

#### 2.2.3. Sharpe Ratio Model

The Sharpe ratio model is another portfolio selection model which includes the ratio of profit ξ(E) and risk υ(E). The objective is to maximize the ratio, which is given as,

(23) $$\begin{array}{c} SR=\frac{\xi (E)}{\upsilon (E)}. \end{array}$$

We will inverse the formulation (23) to form a minimization problem. The Sharpe ratio model will help to avoid the additional hyper-parameter λ. Now, the objective is to minimize $\frac{\upsilon (E)}{\xi (E)},$ which will in turn maximize the profit and minimize the risk. Again, we can formulate the model using (2), (3), and (11)–(13), which is given as,

$$\begin{array}{cc} \mathrm{Minimization}:\; & \end{array}$$

(24) $$\begin{array}{cc} \; & \min_{E}\frac{\upsilon (E)}{\xi (E)}\\ \mathrm{Subject}\;\mathrm{to}:\; & \end{array}$$

(25) $$\begin{array}{cc} \; & \chi \left(E\right)=\left(1+\alpha \right)E^{T}=1 \end{array}$$

(26) $$\begin{array}{cc} \; & \sum_{i=1}^{K}b_{i}=n \end{array}$$

(27) $$\begin{array}{cc} \; & 0\leq E\leq b. \end{array}$$

This model will be used for the portfolio optimization. In the next section, we will formulate the Distributed Beetle Antennae Search (DBAS), which we will be used to solve the portfolio problem given in (24)–(27).

## 3. Distributed Beetle Antennae Search (DBAS)

In this section, we will elaborate on the nature of BAS, its formulation, and the algorithm. Later, we will develop DBAS and will discuss its underlying algorithm.

### 3.1. BAS Formulation and Algorithm

BAS has a biologically inspired meta-heuristic algorithm, which is inspired by the food-searching behavior of the beetle. It has two antennas, i.e., left $X_{l}$ and right $X_{r}.$ It registers the smell of the food on both antennae, and based on the intensity of the smell, it either moves in the left direction or right. The beetle repeats this procedure iteratively until it finally reaches the food source. Details on the behavior of beetles and BAS are presented in [56]. Here, we will look into updating the equations of the BAS algorithm, which are responsible for converging the searching particle toward the optimal solution.

Imagine a non-linear objective function F(X), where X is a vector of optimizing variables. We can represent an objective function as,

(28) $$\begin{array}{c} \min_{X}F\left(X\right). \end{array}$$

Here, we are solely considering an objective function, while the real-world problem would include additional constraints, as shown in (24)–(27). The objective of the BAS is to find the optimal solution of (28) by tweaking the values of X such that the maximum value of (28) is achieved. Here, it is worth mentioning that we can assume the maximization problem as well. The formulation will be a little different, which will be shown later during the derivation.

From this point forward, we will refer to the beetle as a particle. Let us say that the current position of the particle is $X\in R^{1\times K}.$ To compute the new position of the particle, we will move it slightly to the right $X_{r}$ and slightly to the left $X_{l},$ which is given as

(29) $$\begin{array}{cc} X_{r} & =X+db \end{array}$$

(30) $$\begin{array}{cc} X_{l} & =X-db, \end{array}$$

where $b\in R^{1\times K}$ is similar to X. Here, *d* is an antenna length, which determines how big or small a step should be taken in each iteration. In the beginning, *d* will have a larger value allowing the particle to explore more of the search space, and over time when the particle approaches the optimal solution, the value of *d* becomes small.

The next step is to compute the objective function value at $X_{r}$ and $X_{r},$ so that BAS can decide its course, i.e., either in the $X_{r}$ direction or $X_{l}$ direction. The computation of F(X) is given as,

(31) $$\begin{array}{cc} F_{r} & =F(X_{r}) \end{array}$$

(32) $$\begin{array}{cc} F_{l} & =F(X_{l}), \end{array}$$

where $F_{r}$ and $F_{l}$ are scalar values of the objective function at $X_{r}$ and $X_{l}$ respectively. Now, BAS will compute the next step based on the difference of the values of $F_{r}$ and $F_{l},$ which is given as,

(33) $$\begin{array}{cc} \Delta F & =F_{r}-F_{l} \end{array}$$

(34) $$\begin{array}{cc} X & =X-\tau \mathrm{sgn}(\Delta F)b, \end{array}$$

where sgn is an activation function to translate the value of ΔF between [−1,1]. Likewise, τ is another hyper-parameter, which is used to decide the step length of the BAS particle. This concludes the general framework of BAS. The algorithm is shown in Algorithm 1.
**Algorithm 1** BAS Algorithm.**Objective Function:**F(X,O)**DBAS Initialization:**η=0.99d=0.9τ=0.9**Portfolio Initialization:**T=iterD=dimX=randn(1,D)$O=[M_{n},C_{n}]$$M_{n}:$ Mean $C_{n}:$ Covariance$F_{\mathrm{best}}=F\left(X,P\right)$$X_{\mathrm{best}}=X$**For**i=1:T       b=randn(1,D)       Compute $X_{r}$ and $X_{l}$ using (29) and (30)       Compute $F_{r}$ and $F_{l}$ suing (31) and (32)       Compute X using (34)       F=F(X,O)              **If**$F_{min}<F_{best}:$               $F_{best}=F_{max}$               $X_{\mathrm{best}}=X$       **Else:**              $X=X_{\mathrm{best}}$       **End If**              d=ηd+0.01       τ=ητ**End For**

This algorithm is designed for the portfolio selection problem. The objective function includes F(X,O), where O includes all the parameters necessary for the portfolio problem, i.e., M means of the stocks, C covariance matrix of the stocks, and other constraints. The single-particle BAS algorithm is limited to single portfolio optimization because of its computational limitations. In order to optimize multiple portfolios at once, we need to introduce a more robust nature in the already existing framework, which we will introduce in DBAS formulation.

**Remark** **1.** *The above formulation of BAS is for the minimziation problem. For the maximization problem, we need to modify the (34), as shown below,*

(35) $$\begin{array}{c} X=X+\tau \mathrm{sgn}(\Delta F)b, \end{array}$$

*where the − sign is replaced with the + sign. It will help BAS to move up the valley in the search space.*

**Figure 2:**
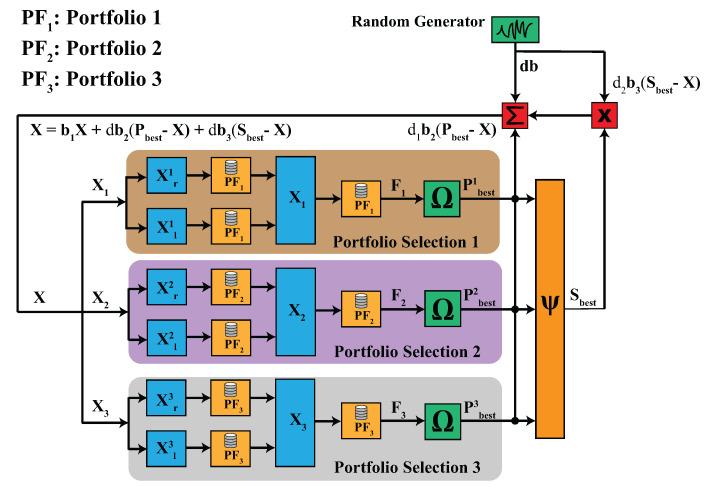
The schematic of the DBAS algorithm for the multi-portfolio selection problem. It consists of 3 portfolios and clearly shows that each particle $\left[X_{1},X_{2},X_{3}\right]$ optimizes portfolio $PF_{1},PF_{2},$ and $PF_{3}$, respectively. It can be seen that particles do not share any portfolio database or stock information among the swarm, which ensures privacy.

In the next section, we will formulate the DBAS algorithm. Unlike BAS, the DBAS formulation will be better focused on the portfolio problem. However, it is worth mentioning that the algorithm is not problem-specific, as an objective function must be defined for any real-world problem. The schematic of the proposed algorithm is shown in Figure 2.

### 3.2. DBAS Formulation and Algorithm

DBAS is a hybrid variant of BAS and PSO (Particle Swarm Optimization) algorithms. It mimics the swarm-like nature of PSO, where each step of the particles is based on the particle’s best position $P_{\mathrm{best}}$ and the swarm’s best position $S_{\mathrm{best}}$. However, it uses the BAS updating strategy given in (34).

#### 3.2.1. DBAS Updating Criteria

We will modify the updating criteria for $X_{r}$ and $X_{l}$ given in (29) and (30). The updating criteria depend on three factors: the particle’s best position $P_{\mathrm{best}},$ the swarm’s best solution $S_{\mathrm{best}},$ and the random direction db. The modified updating criteria is given as,

(36) $$\begin{array}{cc} V & =b_{1}V+d_{1}b_{2}\left(P_{\mathrm{best}}-X\right)+d_{2}b_{3}\left(S_{\mathrm{best}}-X\right) \end{array}$$

(37) $$\begin{array}{cc} X_{r} & =X+db+V \end{array}$$

(38) $$\begin{array}{cc} X_{l} & =X-db-V, \end{array}$$

where $b\in R^{D\times K}$ and $[b_{1},b_{2},b_{3}]\in R.$ Here, $X\in R^{D\times K}$ represents the particles positions, $P_{best}\in R^{D\times K}$ shows the particles best positions, and $S_{\mathrm{best}}\in R^{D\times K}$ is the swarm’s best position, which are stacked in a row to meet the dimensionality requirement. Likewise, $\left[d,d_{1},d_{2}\right]$ are scalars. Typical swarm algorithms are used to compute a single objective function, but our objective is to optimize multiple objective functions since each portfolio comprises different stocks. Therefore, it is worth mentioning that $X\in R^{D\times K},$ where *D* and *K* represent the number of portfolios and portfolio size, respectively. Likewise, $\left\{b_{1},b_{2}\right\}\in R^{D\times K}$ are two random vectors and *d* represents the antenna size. Each particle in DBAS corresponds to a single portfolio. The objective is that each particle will optimize its corresponding portfolio locally, i.e., $P_{best}^{j}\in R^{1\times K},$ where j∈{1,2,3,⋯,D}, and the swarm will optimize all portfolios, i.e., $S_{\mathrm{best}},$ collectively.

After computing $X_{r}$ and $X_{l},$, the next step is to compute the objective function value for each portfolio, i.e., each particle. It is given as,

(39) $$\begin{array}{c} F_{r}=F\left(X_{r},O\right) \end{array}$$

(40) $$\begin{array}{c} F_{l}=F\left(X_{l},O\right), \end{array}$$

where O includes all the parameters necessary for portfolio optimization as mentioned in the problem formulation section. Here, $\left\{F_{r},F_{l}\right\}\in R^{D\times 1}$ and F(.) is a vector of dimension D; it includes the objective functions of all portfolios. Next, each position of particle X is updated using,

(41) $$\begin{array}{cc} \Delta F & =F_{r}-F_{l} \end{array}$$

(42) $$\begin{array}{cc} X & =X-\tau \mathrm{sgn}(\Delta F)b. \end{array}$$

Then, the objective function value of each portfolio is computed, i.e., the particle at X, which is given as,

(43) $$\begin{array}{c} F=F(X,O), \end{array}$$

where F has similar dimensions as $F_{r}$ and $F_{l}.$ If the objective function value $F_{j}\left(.\right)$ of a *j*-th particle at $X_{j}\in R^{1\times K}$ is less than its personal best $P_{\mathrm{best}}^{j},$ then update $P_{\mathrm{best}}^{j}$ with $X_{j},$. Otherwise, retain the old best values. This updating rule for all the particles X is given as,

(44) $$\begin{array}{c} P_{\mathrm{best}}=\Omega \left(X\right)=\left\{\begin{array}{cc} X, & \mathrm{if}\;F(X)<P_{\mathrm{best}}\\ P_{\mathrm{best}}, & \mathrm{otherwise}. \end{array}\right. \end{array}$$

Now, the next step is to obtain the swarm’s best position. This can be done by simply looking for the minimum value of the objective function in F(.), which is given as,

(45) $$\begin{array}{c} {}[F,j]=\min(F(X,O)). \end{array}$$

If the value of *F* is less than the swarm’s best solution $G_{best},$ then update $S_{\mathrm{best}};$ otherwise, retain the old best, which is given as,

(46) $$\begin{array}{c} S_{\mathrm{best}}=\psi \left(F\right)=\left\{\begin{array}{cc} X_{j}, & \mathrm{if}\;F<G_{best}\\ S_{\mathrm{best}}, & \mathrm{otherwise}. \end{array}\right. \end{array}$$

To further randomize the process, we have included another condition to the update *d* and τ. We have included a random variable r=rand∈[0,1]; if r<0.5, then we will update the values of *d* and τ. The pseudocode is shown in Algorithm 2.
**Algorithm 2** DBAS Algorithm.**Objective Function:**F(X,O)**DBAS Initialization:**η=0.99d=0.9$d_{1}=0.9$$d_{2}=0.9$τ=0.9**Portfolio Initialization:**T=iter        %IterationsD=pop        %Total PortfoliosK=dim        %No. of Stocks In Each PortfolioX=randn(D,K)$X_{\mathrm{best}}=X$$O=[O_{1},O_{2},O_{3},\cdots ,O_{K}]$% $O_{K}$ Includes Parameters For *k*-th Portfolio% $O_{K}=\left[M_{K},C_{K}\right]$$M_{K}:$ Mean $C_{K}:$ Covariance$P_{\min}=F\left(X,P\right)$$\left[G_{best},\mathrm{ind}\right]=\min(F\left(X,P\right)$$S_{best}=X_{\mathrm{ind}}$**For**i=1:T       b=randn(D,K)       $b_{1}=\mathrm{randn}\left(1,1\right)$       $b_{2}=\mathrm{randn}\left(1,1\right)$       $b_{3}=\mathrm{randn}\left(1,1\right)$       Compute V using (36)       Compute $X_{r}$ and $X_{l}$ using (37) and (38)       Compute $F_{r}$ and $F_{l}$ suing (39) and (40)       Compute X using (42)       **For**j=1:D              $F=F_{j}\left(X_{j},O_{j}\right)$              **If**$F<P_{min}^{j}:$                     $P_{min}^{j}=F$                     $P_{best}^{j}=X_{j}$              **End If**                     **End For**              [F,ind]=min(F(X,O))              **If**$F<G_{best}:$              $G_{best}=F$              $s_{\mathrm{best}}=X_{\mathrm{ind}}$              $S_{\mathrm{best}}=\mathrm{repmat}\left(s_{\mathrm{best}},D,1\right)$       **End If**       r= rand       **If**r<0.5:              d=ηd+0.01              $d_{1}=\eta d_{1}+0.01$              $d_{2}=\eta d_{2}+0.01$              τ=ητ       **End If**              **End For**

#### 3.2.2. DBAS Privacy Policy

**Figure 3:**
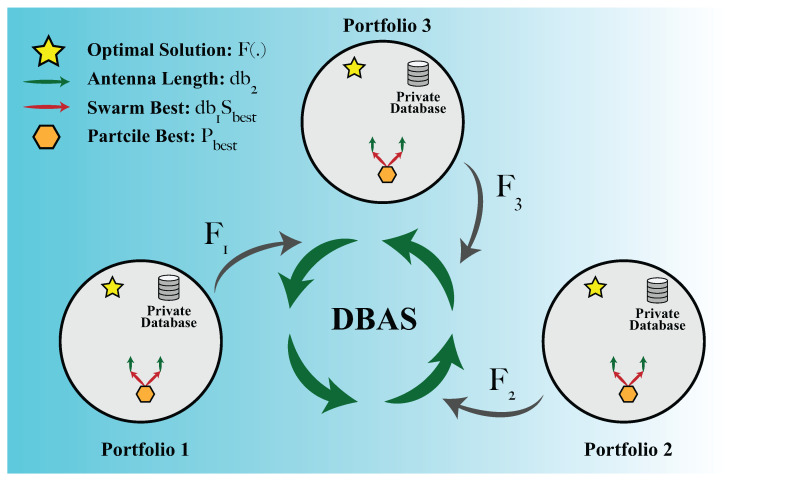
Each particle in DBAS is assigned to a single portfolio, and the particle alone has access to the private database of the portfolio. In DBAS, the particle in swarm only shares the objective function values F, i.e., gradients.

Each particle in DBAS deals with a single portfolio. A particle alone has access to the private data of the assigned portfolio, including: stock names, investment amount, mean of the portfolio, and variance of the portfolio. The portfolio is like a black box. The particle $X_{j}$ will enter the black box $F_{j}\left(X_{j},O\right)$, will compute the objective function of the portfolio and will give out the objective function value $F_{j}$ while keeping the privacy of the private database and portfolio information. The objective function value, i.e., gradient, will be available to all the other particles and based on all the gradients, i.e., objective function values of all particles, the DBAS algorithm will compute the swarm’s best solution to decide the next step. It helps the DBAS to efficiently find the optimal selection of all portfolios without violating the confidentiality and privacy of portfolios. The concept of DBAS privacy is shown in Figure 3.

## 4. Simulation Results

In this section, we will discuss the stock data used for the multiple portfolio selection problem. Furthermore, the DBAS algorithm will be employed to solve the optimization problem formulated in the problem formulation selection.

### 4.1. Stock Data

**Figure 4:**
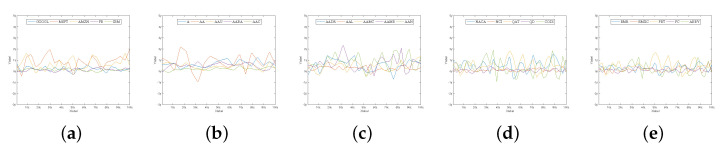
The return rate for all the 25 companies for 200 days of NASDAQ stock data in 2017. (**a**–**e**) represents the data of companies 1–5, companies 6–10, companies 11–16, companies 16–20, and companies 20–25, respectively.

For the simulation, we collected real-world stock data from the NASDAQ stock market in 2017. The data comprise several hundred stock companies. A total of 25 known companies were selected for the simulation. Then, 200 days worth of stock closing prices were collected for each company to understand the data trend in more detail and to provide more robust portfolios. All the 25 companies are shown in Table 2. The volatility of the stock market is shown in Figure 4. It can be seen how abruptly the stock prices (return rate) change over time.

In our simulation, there are four multi-portfolio optimization problems, i.e., 5 stock companies, 10 stock companies, 15 stock companies, and 20 stock companies. In each multi-portfolio selection problem, we will be optimizing *three* portfolios. For instance, in the *five* stock companies multi-portfolio selection problem, DBAS will optimize three portfolios, each looking for an optimal selection of stock companies. Considering the stochastic nature of the DBAS algorithm and how results can vary with each experiment, we performed simulation on each portfolio for 10 times, and the average or mean result is obtained. The computational resources used in the simulations are given below:

- Software: MATLAB;
- System: MacBook Pro;
- Processor: 2.2 GHz;
- Cores: 6–Core Intel Core i7;
- Memory: 6 GB 2400 MHz DDR4;
- Graphics: Radeon Pro 555X 4 GB.

### 4.2. Three Portfolios of Five Stock Companies

For the first simulation category, *three* portfolios were selected, each consisting of *five* companies. The objective is to optimize the three objective functions (24)–(27), i.e., portfolios, such that the raw data of each portfolio remain private and without sharing the private databases that DBAS optimizes. There are a few hyper-parameters in DBAS, i.e., antenna length *d* and particle step-size τ. We selected the values of these hyper-parameters through trial and error, e.g., τ=0.900,d=0.999, and η=0.900, which are shown in Table 3. In addition, a few other parameters were selected for the simulation, i.e., total iterations T=500, swarm population D=3, dimension of each portfolio K=5.

**Figure 5:**
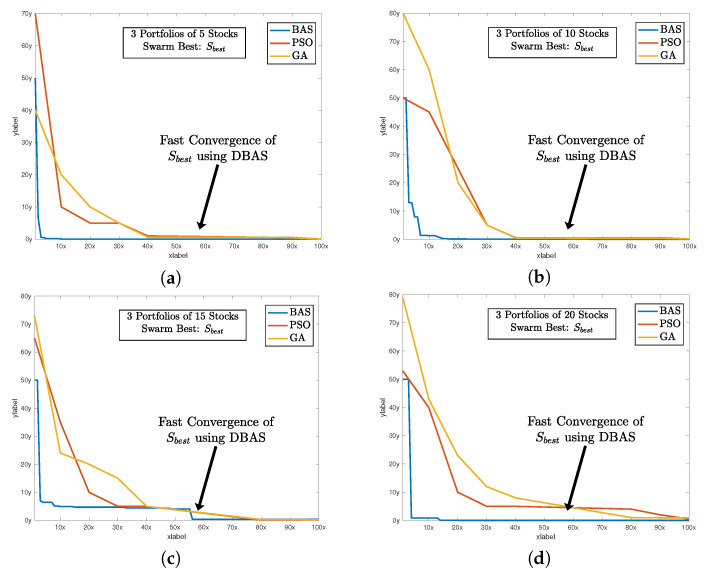
The sharp and fast convergence of DBAS swarm’s $G_{best}$ toward the minima while selecting optimal configuration of all 3 portfolios. It also shows the comparison with PSO and GA algorithms, and it can be seen that the convergence profile of DBAS is fast compared to others. (**a**) shows the global convergence profile of all algorithms. Likewise, (**b**) shows the convergence of BAS, PSO, and GA. And finally, (**c**,**d**) shows the convergence of 15 and 20 stocks of all three algorithms respectively.

**Figure 6:**
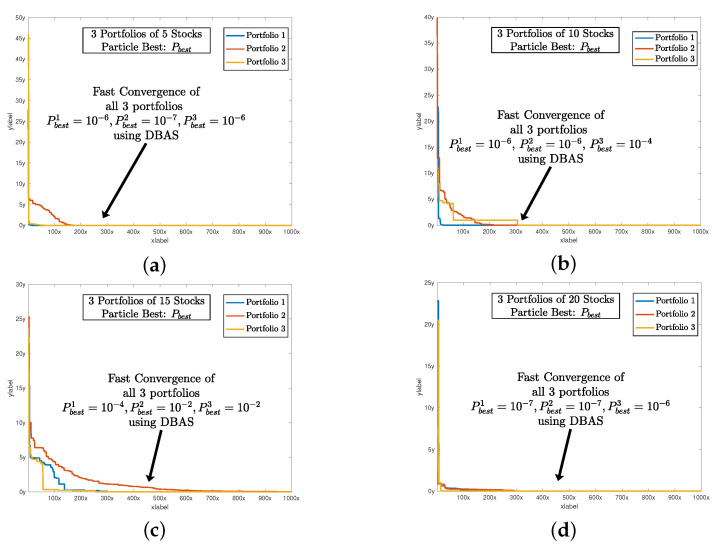
The sharp and fast convergence of DBAS particles $P_{best}$ toward their respective minimas while selecting an optimal configuration of their portfolios. Since we are dealing with three portfolios, we can see the three convergence profiles. (**a**) shows the global convergence profile of all algorithms. Likewise, (**b**) shows the convergence of BAS, PSO, and GA. And finally, (**c**,**d**) shows the convergence of 15 and 20 stocks of all three algorithms respectively.

**Figure 7:**
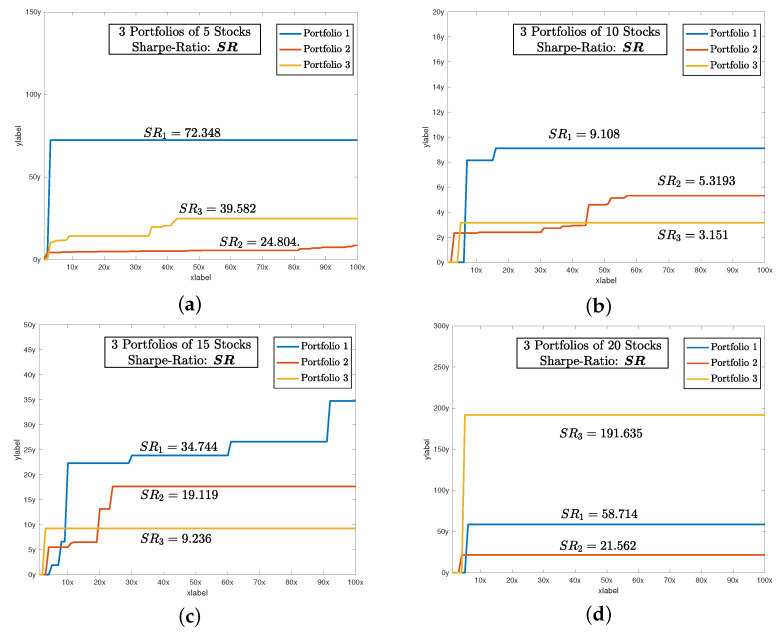
The rising trend of Sharpe ratio SR as the DBAS moves towar the optimal solution of portfolio selection problem. The higher value of SR indicates that each portfolio has a higher profit ξ(E) and lower risk υ(E), as shown in (23). (**a**) shows the convergence profile of sharpe-ratio of 5 stocks. Likewise, (**b**) shows the convergence of sharpe-ratio of 10 stocks. And finally, (**c**,**d**) shows the convergence of sharpe-ratio of 15 and 20 stocks respectively.

**Figure 8:**
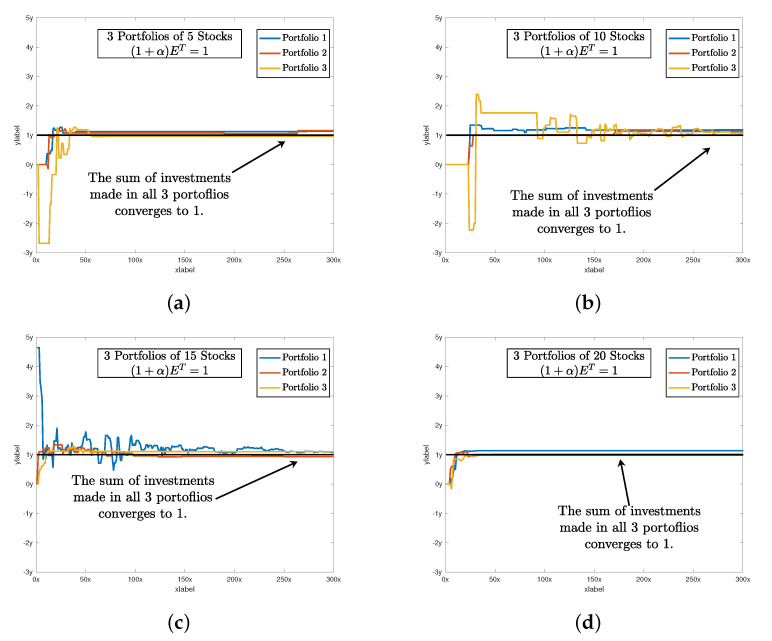
It shows that as the portfolio selection problem converges toward the optimal solution, the sum of normalized investment (1+α)E≈1. There are some spikes in the beginning; however, all the particles of the respective portfolio converge to 1. (**a**) shows the convergence of normalized investment (1+α)E≈1 of 5 stocks. Likewise, (**b**) shows the convergence of normalized investment of 10 stocks. And finally, (**c**,**d**) shows the convergence of normalized investment of 15 and 20 stocks respectively.

After setting all of the parameters. DBAS is ready to optimize the portfolio problem mentioned in (24)–(27). The simulation results are evaluated based on three parameters: swarm convergence $G_{best},$ particles convergence $P_{\mathrm{best}},$ Sharpe ratio SR, and the total investment constraint (1+α)E=1. The results are shown in Table 3 and the convergence profiles of $G_{best},P_{\mathrm{best}},SR$ and (1+α)E=1 are shown in Figure 5, Figure 6, Figure 7 and Figure 8a. For three portfolios of five stock companies, the swarm best solution turns out to be $10^{-7}$, and the convergence profile in Figure 5a shows how efficiently and quick DBAS converges to the optimal solution. Likewise, the convergence of each particle, i.e., each portfolio, is also robust, as shown in Figure 6a. However, the convergence rates do not give us the relationship between the profit and risk. For that, the Sharpe ratio curve was computed. It can be seen in Figure 7a that each portfolio has achieved substantial profit with minimal risk involved. Finally, it is essential that the investment in stocks remains around ≈1. We have shown the convergence profile of (1+α)E=1 in Figure 8a, and it can be seen that all three investments converge to around 1, which is required. The detailed results are also shown in Table 3.

The main takeaway from the results is that DBAS can converge multi-portfolios and ensures the clients’ secrecy and privacy. In the simulated results, it can be seen that each particle in DBAS only shares the objective function value $P_{best}^{j},$ i.e., gradient with other particles, whereas the private information of the portfolio remains inaccessible to other portfolios.

### 4.3. Three Portfolios of 10 Stock Companies

For the second simulation scenario, we again selected *three* portfolios, each consisting of 10 companies. The values of hyper-parameters were once again chosen through trial and error, which are also shown in Table 3. In addition, the other parameters for the simulation include total Iterations T=500, swarm population D=3, dimension of each portfolio K=10.

The simulation results are shown in Table 3, and the convergence profiles of $G_{best},P_{\mathrm{best}},SR$ and (1+α)E=1 are shown in Figure 5, Figure 6, Figure 7 and Figure 8b. For the three portfolios of 10 stock companies, the swarm best solution turns out to be $10^{-6}.$ Its convergence profile is shown in Figure 5b. Likewise, the convergence of each particle, i.e., each portfolio, is also robust and shown in Figure 6b. Their convergence values turn out to be optimal, i.e., $P_{\mathrm{best}}=\left[10^{-6},10^{-6},10^{-4}\right].$ The Sharpe ratio curve is shown in Figure 7b, and it can be seen that each portfolio has achieved substantial profit with minimal risk. We have also shown the convergence profile of (1+α)E=1 in Figure 8b, where it can be seen that all three investments converge to around 1, which is again required. The detailed results are also shown in Table 3.

### 4.4. Three Portfolios of 15 Stock Companies

For the third simulation scenario, we again selected *three* portfolios, each consisting of 15 companies. Once again, the values of the hyper-parameters were chosen through trial and error, which are also shown in Table 3.

The simulation results are shown in Table 3 and the convergence profiles of $G_{best},P_{\mathrm{best}},SR$ and (1+α)E=1 are shown in Figure 5, Figure 6, Figure 7 and Figure 8c. For the three portfolios of 15 stock companies, the swarm’s best solution turns out to be $10^{-4}.$ Its convergence profile is shown in Figure 5c. Likewise, the convergence of each particle, i.e., each portfolio, is also robust and shown in Figure 6c. Their convergence values turn out to be again optimal, i.e., $P_{\mathrm{best}}=\left[10^{-4},0.020,0.001\right].$ The Sharpe ratio curve is shown in Figure 7b, and it can be seen that each portfolio has again achieved substantial profit with minimal risk. We have also shown the convergence profile of (1+α)E=1 in Figure 8c, and it can be seen that all three investments converge to around 1, which is required. The detailed results are also shown in Table 3.

### 4.5. Three Portfolios of 20 Stock Companies

For the fourth and the last simulation scenario, we selected *three* portfolios, each consisting of 20 stock companies. Hyper-parameters are also shown in Table 3.

The simulation results are shown in Table 3 and the convergence profiles of $G_{best},P_{\mathrm{best}},SR$ and (1+α)E=1 are shown in Figure 5, Figure 6, Figure 7 and Figure 8d. For three portfolios of 15 stock companies, the swarm’s best solution turns out to be $10^{-7}.$ Its convergence profile is shown in Figure 5d. Likewise, the convergence of each particle, i.e., each portfolio, is also robust and shown in Figure 6d. Their convergence values turns out to be optimal, i.e., $P_{\mathrm{best}}=\left[10^{-7},10^{-7},10^{-6}\right].$ The Sharpe ratio curve is shown in Figure 7b, and it can be seen that the ratio is again substantially high. We have also shown the convergence profile of (1+α)E=1 in Figure 8d, and it can be seen that all three investments converge to around 1, which is required. The detailed results are also shown in Table 3.

The primary limitation of DBAS is the “Virtual particle,” which means that for each iteration, we have to compute the objective function value for three times for $F_{r},F_{l},$ and F. It could make it computationally expensive and consume time. In the future, we will work on eliminating the “virtual particle” limitation, and we will also include some other meta-heuristic algorithms such as GSK [57] to compare with. Likewise, we will work on some additional statistical analysis including non-parametric statistical tests such as Wilcoxon Signed Rank Test and Friedman.

## 5. Conclusions

In this paper, a framework to ensure the privacy of stock portfolios in the multi-portfolio selection problem was proposed. This involved the design of a distributed variant of the BAS algorithm known as Distributed Beetle Antennae Search (DBAS). DBAS combines the swarm-like behavior of PSO with BAS’s updating rule. Each particle in the swarm is assigned to optimize a single portfolio, and the particles only share the gradient of their respective portfolio with the swarm, avoiding the exposure of the private database and stock information of portfolios. The algorithm was then simulated on real-world stock data extracted from the NASDAQ stock market for 25 stock companies. The simulation is divided into *four* categories, and in each category, *three* portfolio models were optimized. The simulation results show that DBAS not only ensures the privacy of portfolios but is also robust, computationally economical, and time efficient.

## Figures and Tables

**Table 1:** Hyper-Parameters.

Name	Notation	Values (Default)
Antennae Length	*d*	0.9
Step Size	τ	0.9
Personal Antennae Length	$d_{1}$	0.9
Global Antennae Length	$d_{2}$	0.9
Step Multiplier	η	0.99

**Table 2:** NASDAQ Symbols of The Stock Companies In Portfolio Selection.

Companies 1–5	GOOGL	MSFT	AMZN	FB	IBM
**Companies 6–10**	A	AA	AAU	AABA	AAC
**Companies 11–15**	AADR	AAL	AAMC	AAME	AAN
**Companies 16–20**	NCA	NCI	QAT	QD	CODI
**Companies 20–25**	EMR	EMXC	FET	FC	ABBV

**Table 3:** DBAS Convergence Profile for Different Portfolios.

		Companies: 5			Companies: 10	
	**Portfolio 1**	**Portfolio 2**	**Portfolio 3**	**Portfolio 1**	**Portfolio 2**	**Portfolio 3**
*n*	5	5	5	7	8	9
$G_{best}$	$10^{-7}$	$10^{-7}$	$10^{-7}$	$10^{-6}$	$10^{-6}$	$10^{-6}$
$P_{\mathrm{best}}$	$10^{-6}$	$10^{-7}$	$10^{-6}$	$10^{-6}$	$10^{-6}$	$10^{-4}$
SR	72.348	24.804	39.582	9.108	5.319	3.151
(1+α)E=1	1.116	1.179	1.065	1.153	1.140	0.964
**DBAS:**						
η		0.900			0.900	
$d=d_{1}=d_{2}$		0.999			0.900	
τ		0.900			0.900	
sgn		[−1,1]			[−1,1]	
**Some Additional Parameters:**						
T		500			500	
D		3			3	
K		5			5	
H(E,σ) Eval.		4500			4500	
		**Companies: 15**			**Companies: 20**	
	**Portfolio 1**	**Portfolio 2**	**Portfolio 3**	**Portfolio 1**	**Portfolio 2**	**Portfolio 3**
*n*	15	12	10	15	20	18
$G_{best}$	$10^{-4}$	$10^{-4}$	$10^{-4}$	$10^{-7}$	$10^{-7}$	$10^{-7}$
$P_{\mathrm{best}}$	$10^{-4}$	0.020	0.001	$10^{-7}$	$10^{-7}$	$10^{-6}$
SR	7.088	3.667	4.667	58.71	21.56	191.6
(1+α)E=1	0.930	1.088	1.103	0.9893	1.136	1.021
**DBAS:**						
η		0.850			0.950	
$d=d_{1}=d_{2}$		0.900			0.900	
τ		0.850			0.850	
sgn		[−1,1]			[−1,1]	
**Some Additional Parameters:**						
T		500			500	
D		3			3	
K		5			5	
H(E,σ) Eval.		4500			4500	

## Data Availability

Not applicable.
